# Intestinal FFA2 promotes obesity by altering food intake in Western diet-fed mice

**DOI:** 10.1530/JOE-23-0184

**Published:** 2024-01-11

**Authors:** Kristen R Lednovich, Sophie Gough, Medha Priyadarshini, Nupur Pandya, Chioma Nnyamah, Kai Xu, Barton Wicksteed, Sidharth Mishra, Shalini Jain, Joseph L Zapater, Jose Cordoba-Chacon, Hariom Yadav, Brian T Layden

**Affiliations:** 1 Division of Endocrinology, Diabetes and Metabolism, Department of Medicine, University of Illinois at Chicago, Chicago, Illinois, USA; 2 USF Center for Microbiome Research, University of South Florida Morsani College of Medicine, Tampa, Florida, USA; 3 Jesse Brown Veterans Affairs Medical Center, Chicago, Illinois, USA

**Keywords:** FFA2, free fatty acid receptor 2, short-chain fatty acid receptor, gut microbiota, obesity, metabolic homeostasis, food intake

## Abstract

Short-chain fatty acids (SCFAs) are key nutrients that play a diverse set of roles in physiological function, including regulating metabolic homeostasis. Generated through the fermentation of dietary fibers in the distal colon by the gut microbiome, SCFAs and their effects are partially mediated by their cognate receptors, including free fatty acid receptor 2 (FFA2). FFA2 is highly expressed in the intestinal epithelial cells, where its putative functions are controversial, with numerous *in vivo* studies relying on global knockout mouse models to characterize intestine-specific roles of the receptor. Here, we used the Villin-Cre mouse line to generate a novel, intestine-specific knockout mouse model for FFA2 (Vil-FFA2) to investigate receptor function within the intestine. Because dietary changes are known to affect the composition of the gut microbiome, and can thereby alter SCFA production, we performed an obesogenic challenge on male Vil-FFA2 mice and their littermate controls (FFA2-floxed, FFA2^fl/fl^) to identify physiological changes on a high-fat, high-sugar ‘Western diet’ (WD) compared to a low-fat control diet (CD). We found that the WD-fed Vil-FFA2 mice were transiently protected from the obesogenic effects of the WD and had lower fat mass and improved glucose homeostasis compared to the WD-fed FFA2^fl/fl^ control group during the first half of the study. Additionally, major differences in respiratory exchange ratio and energy expenditure were observed in the WD-fed Vil-FFA2 mice, and food intake was found to be significantly reduced at multiple points in the study. Taken together, this study uncovers a novel role of intestinal FFA2 in mediating the development of obesity.

## New and noteworthy

A novel intestine-specific knockout mouse model for FFA2 (Vil-FFA2) was used to investigate the *in vivo* role of this receptor in the intestine. Male Vil-FFA2 mice and floxed littermate controls were challenged with a high-fat high-sugar Western diet (WD) for 25 weeks and comprehensively characterized. We found that WD-fed Vil-FFA2 mice were protected from obesity and hyperglycemia; however, this effect was lost by the end of the study. Furthermore, the WD-fed Vil-FFA2 mice consumed significantly less food when compared to the WD-fed controls, indicating that phenotypic differences are driven by changes in food intake. These data reveal an important role of intestinal FFA2 in contributing to the development of diet-induced obesity.

## Introduction

Free fatty acid receptor 2 (FFA2) is a G protein-coupled receptor belonging to the short-chain fatty acid family of receptors. The endogenous ligands for FFA2, short-chain fatty acids (SCFAs), are a group of key metabolites that are crucial to maintaining metabolic homeostasis (Le Poul *et al.* 2003). The gut microbiome is the major generator of SCFAs, which are synthesized primarily in the distal colon via bacterial fermentation of dietary fibers, where they carry out an expansive repertoire of functional roles including the regulation of glucose, lipid, and energy metabolism, and have localized effects on intestinal function within the gastrointestinal tract (Macfarlane & Macfarlane 2012, Tan *et al.* 2014). Additionally, SCFAs act as major mediators of crosstalk between the gut microbiome and human host, as fluctuating levels are influenced by changes in gut microbial composition and largely impacted by dietary components (Ríos-Covián *et al.* 2016). One way that SCFAs are able to carry out their physiological effects is through binding to their cognate receptors, including FFA2.

FFA2 is broadly expressed in many metabolically active tissues, including the intestine, endocrine pancreas, adipose tissue and immune cells, where it collectively mediates the diverse set of functions facilitated by SCFAs (Priyadarshini *et al.* 2018). In both mice and humans, FFA2 has the highest binding affinity for acetate, followed by propionate and butyrate, and has been reported to signal via multiple Gα pathways, including the excitatory Gα_q/11_ pathway and the inhibitory Gα_i/o_ pathway (Engelstoft *et al.* 2013, McNelis *et al.* 2015). Within bone marrow-derived immune cells, the receptor has been found in regulatory T cells, macrophages, and monocytes (Cox *et al.* 2009). In these cell types, FFA2 plays an important role in mediating the immunogenic effects of SCFAs and has been implicated in a variety of processes, including inflammation and responses to infection and disease (Maslowski *et al.* 2009, Masui *et al.* 2013, Kamp *et al.* 2016). FFA2 has also been characterized within the pancreatic β-cell, where it promotes the secretion of insulin as well as regulates β-cell mass to contribute to whole-body glucose homeostasis (Fuller *et al.* 2015, Priyadarshini *et al.* 2015, Villa *et al.* 2016). Within the adipose tissue, FFA2 has been reported to inhibit insulin-mediated fat accumulation while enhancing energy expenditure and systemic insulin sensitivity through unclear mechanisms (Kimura *et al.* 2013).

FFA2 is highly expressed within the intestinal epithelium, which is immediately adjacent to where SCFAs are produced (Dass *et al.* 2007). Previous studies have reported the expression of FFA2 specifically within enteroendocrine cells (EECs) and intestinal epithelial cells (IECs) (Karaki *et al.* 2006, Andersen *et al.* 2018). Within EECs, FFA2 has been suggested to mediate the release of multiple postprandial peptide hormones, including GLP-1 and peptide tyrosine tyrosine (PYY), which together modulate glucose metabolism, intestinal transit, and satiety (Psichas *et al.* 2005, Karaki *et al.* 2008, Tolhurst *et al.* 2012). There is a large body of conflicting data regarding the ability of FFA2 to mediate the release of enteroendocrine hormones; therefore, these roles are still considered controversial. Furthermore, FFA2 may influence the secretion of other gut peptide hormones, and several studies have suggested a role in the secretion of the hormones 5-hydroxytryptamine (5-HT) and gastric inhibitor polypeptide (GIP) (Iwasaki *et al.* 2015, Akiba *et al.* 2017).

FFA2 expression within IECs has been reported to play a role in mediating intestinal inflammation through multiple processes including gut barrier maintenance, recruitment of immune cells, and production of cytokines (Kim *et al.* 2013, Nowarski *et al.* 2015, Greenwood-Van *et al.* 2017). While the role of FFA2 within immune cells has been relatively well characterized, the contributions of FFA2 within intestinal cells to immunogenic processes are still unknown. Experimental limitations, including the use of global knockout mouse models to characterize tissue-specific effects, have contributed to a growing body of conflicting data, and tissue-specific models will be crucial in delineating the precise roles of FFA2 within the intestine.

Previously, we generated and characterized an intestine-specific knockout mouse model for free fatty acid receptor 3 (FFA3), which belongs to the same family of receptors as FFA2 (Lednovich *et al.* 2022). Using the same strategy, we have produced a novel, intestine-specific knockout mouse model for FFA2, Villin-Cre-FFA2 (Vil-FFA2). Because dietary changes are known to affect the composition of the gut microbiome, and thereby SCFA levels, and because FFA2 has been implicated in conditions of metabolic stress, we performed an obesogenic challenge on male Vil-FFA2 mice and FFA2-floxed littermate controls (FFA2^fl/^ ^fl^). Mice were either placed on a high-fat, high-sugar Western diet (WD) or a low-fat control diet (CD) and a comprehensive assessment of physiological and metabolic function was performed to probe for alterations in the absence of the receptor. Our strategy uncovered novel insights into the contribution of intestinal FFA2 to the genesis of obesity through altered food intake, further underscoring its importance in mediating metabolic homeostasis.

## Materials and methods

### Animals

Animal studies were conducted with the approval of the University of Illinois at Chicago (UIC) Institutional Animal Care and Use Committee (IACUC) and in accordance with the recommendations of the Guide for the Care and Use of Laboratory Animals of the National Institutes of Health. *Ffar2* floxed mice (*ffar2^fl/^ ^fl^)* were generated at the Transgenic and Targeted Mutagenesis Laboratory (Northwestern University, Chicago, IL), and Villin-cre mice were obtained from Jackson Laboratory (JAX stock #004586). Mice were group-housed at the Biological Resources Facility at UIC under standard temperature and humidity conditions with a 12-h light–12-h darkness cycle. Mice had access to standard rodent chow diet (Teklad LM-485, Envigo, Indianapolis, IN, USA) *ad libitum*. For the obesogenic challenge experiment, mice received either a low-fiber control diet (CD) (Custom Product #D19010907, Research Diets, Inc., New Brunswick, NJ, USA) or a low-fiber Western diet (WD) (Custom Product #D19010908, Research Diets) beginning at 10 weeks of age. Both diets were customized as previously described (Lednovich *et al.* 2022). Male mice were utilized in these studies and controlled by the inclusion of male FFA2-floxed littermates.

### Generation of villin-Cre-FFA2 mice

*Ffar2^fl/^^fl^* mice were generated using the Targeted Knockout First, Reporter-tagged Insertion with Conditional Potential strategy by the International Knockout Mouse Consortium. The critical portion of exon 3 in Ffar2 was flanked by loxP sites, with upstream elements including FRT – LacZ – loxP – neo – FRT, to generate the targeted allele of *Ffar2*. The linearized vector was transfected into C57BL/6N embryonic stem cells. Clones with homologous recombination at the target locus were identified and used to generate chimeric mice. Chimeras were crossed with FLP recombinase mice to convert the mutated allele to conditional allele. *Ffar2^fl/^^wt^* were then selected and backcrossed to C57BL/6N mice to generate *Ffar2^fl/^^fl^* mice. Finally, *ffar2^fl/^^fl^* mice were crossed with a well-characterized, intestine-specific Cre recombinase driver *Vil1-Cre* to generate the tissue-specific knockout strain, *Villin-Cre-Ffar2* (El Marjou *et al.* 2004). All breeding in these studies was carried out in the Biological Resource Laboratory at the University of Illinois at Chicago.

### Metabolic assessment

For the chow diet studies, measurements including body weight, blood glucose levels and plasma insulin levels were recorded weekly under both *ad libitum* and fasting conditions from mice beginning at 6 weeks of age and continuing until 20 weeks of age. Fasting measurements were taken after a 16-h, overnight fast. Glucose tolerance tests were performed between 21 and 23 weeks of age. Mice were humanely euthanized at 25 weeks of age.

### Blood glucose measurements

Blood glucose levels were measured via tail vein bleed using a OneTouch UltraMini glucometer (LifeScan, Malvern, PA, USA).

### Plasma insulin measurements

Whole blood was collected in heparinized capillary tubes, centrifuged at 600 ***g*** for 15 min at 4°C and used for measurement of insulin by mouse anti-insulin enzyme-linked immunosorbent assay (ALPCO, Salem, NH, USA).

### Fecal collection and 16S sequencing

Bacterial genomic DNA was extracted from ~100 mg of mouse feces using the Qiagen DNA Stool Mini Kit from fecal samples that were collected at the end of protocol period and stored at -80 °C until use. The gut microbiota composition was analyzed using the Illumina MiSeq platform (Illumina, San Diego, CA, USA) and 16S rRNA approach along with bioinformatics tools previously established in our lab (Nagpal *et al.* 2019, 2020*a*,*b*). The 16S rDNA V4 hypervariable region was amplified using universal primer pairs 515 F (barcoded) and 806 R (Caporaso *et al.* 2012). The unique barcoded amplicons were purified using AMPure magnetic purification beads (Agencourt, Beckman Coulter, CA, USA) and further quantified using the dsDNA HS assay kit (Life Technologies in a Qubit-3 fluorimeter (Invitrogen) and normalized to prepare the amplicon library (Caporaso *et al.* 2012). The library consisting of each amplicon in equal concentration (8 pM) was used for sequencing on an Illumina MiSeq sequencer using MiSeq reagent kit v3. The sequenced bacterial sequences were demultiplexed, quality filtered, clustered, and analyzed by using quantitative insights into microbial ecology 2 (QIIME2) and R-based analytical tools (Nagpal *et al.* 2019, 2020*a*).

### Obesogenic challenge

For the obesogenic challenge studies, mice were fed with standard chow diet until 10 weeks of age. Baseline measurements were recorded at 8–9 weeks of age, and mice were subsequently placed on either CD or WD from 10 to 35 weeks of age. Mice were humanely euthanized at the completion of the study.

### Nuclear magnetic resonance spectroscopy

Body composition, including lean tissue, fat tissue, and free body fluid, was quantified every 4 weeks following dietary intervention. Unanesthetized mice were analyzed in a benchtop LF50 body composition analyzer (BCA) (Bruker Corporation, Billerica, MA, USA).

### Metabolic cages

Following 10 weeks of dietary intervention, mice were individually housed in metabolic cages (Mouse Promethion Continuous caging system; Sable Systems™, Las Vegas, NV, USA). Cages were maintained at a temperature of 22°C on a 14-h light–10-h darkness cycle and mice were acclimated for 24 h prior to data collection. Carbon dioxide (CO_2_) production and oxygen (O_2_) consumption were measured via continuous air-flow sampling. Respiratory exchange ratio (RER) and energy expenditure were calculated as before. Body mass, food intake, and water intake were recorded. For the metabolic cage experiment involving the control diet-fed mice, food and water measurements were manually taken at the beginning and the end of the study. Data are presented as hourly averages for each metabolic parameter, and an ANCOVA analysis was performed with the CalR tool (https://calrapp.org) (Mina *et al.* 2018).

### Food intake

The BioDaq food intake monitoring system (Research Diets) was used to record food intake from each mouse. Mice were individually housed and acclimated to the food intake cages for 5 days before recording. During the 96-h recording period, mice were given access to diet *ad libitum*. Data were analyzed using the corresponding BioDaq DataViewer software.

### Glucose tolerance test

Mice were fasted for 16 h overnight before an intraperitoneally glucose tolerance test (IP-GTT) with 2 g/kg dextrose (Hospira, Lake Forest, IL, USA) or oral glucose tolerance tests (O-GTT) via oral gavage of 2 g/kg dextrose. Blood glucose measurements were taken via tail bleed using a OneTouch Ultramini glucometer (LifeScan, Malvern, PA, USA) at time points 0, 15, 30, 60, and 120 min, and whole blood was also collected in heparinized capillary tubes for insulin measurement at time points 0, 15, and 30 min. For the chow diet study, mice underwent both O-GTTs and IP-GTTs between 21 and 23 weeks of age. For the obesogenic challenge study, an O-GTT was performed prior to dietary intervention at 9 weeks of age, and then again following 14 weeks of dietary intervention.

### Insulin tolerance test

Mice were fasted for 6 h from the beginning of the light cycle. Insulin tolerance tests (ITT) were performed on mice with insulin (Humalog U-100, Eli Lilly, Indianapolis, IN, USA) administered intraperitoneally at a dose of 1.0 U/kg body weight. Blood glucose measurements were taken via tail bleed using a OneTouch UltraMini glucometer (LifeScan) at time points 0, 15, 30, 60, 90, and 120 min.

### Gastrointestinal transit time and fecal measurements

Following 14 weeks on their respective diets, mice were transferred to individual cages prior to the experiment and were singly housed until the completion of the study. A 6% solution of a nonabsorbable dye, carmine red (natural red 4; Sigma-Aldrich) in 0.5% methylcellulose, was prepared and 0.2 mL was administered to each mouse by oral gavage (Welch *et al.* 2014). The cage floor was covered with white paper to facilitate detection of the red dye in feces. The time of gavage was set as *t*_0_. Following gavage, mice were left undisturbed without food and water until the first red fecal pellet appeared (*t*_end_). Gastrointestinal transit time (GI-TT) was calculated as *t*_end_ − *t*_0_. During the first 2 h of the study, all fecal pellets were collected to calculate the number of pellets and fecal pellet weight. Mice that did not produce any pellets were excluded from analysis.

### Tissue isolation and endpoint measurements

For the chow diet study, all mice were sacrificed at 25 weeks of age. For the obesogenic challenge study, all mice were sacrificed at 35 weeks of age. Tissues were removed, weighed if necessary, and then immediately snap frozen in liquid nitrogen for RNA isolation.

### Whole blood sampling and ELISA assays

Following a 4-h fast, mice were administered an oral dextrose bolus and anesthetized via 5% isopropanol inhalation several minutes before euthanization. Mice were then euthanized by cardiac puncture 15 min after administration of oral glucose bolus and whole blood was collected and stored in heparinized blood collection tubes. A cocktail of protease inhibitors including dipeptidyl peptidase-4 inhibitor (Sigma-Aldrich) and aprotinin (Phoenix Pharmaceuticals Inc., Burlingame, CA, USA) was immediately added, and samples were stored on ice for 30 minutes before centrifugation (600 ***g***) at 4°C for 15 min. Plasma portions were isolated for ELISA assays. Plasma GLP-1 levels and GIP levels were quantified by Mouse Total GLP-1 ELISA kit (CrystalChem, Elk Grove Village, IL, USA) and Mouse Total GIP ELISA kit (Millipore), respectively. Lipopolysaccharide (LPS) levels were measured with the murine LPS ELISA kit (Cusabio, Wuhan, China).

### RNA isolation and qPCR

RNA was extracted from tissues using TRIzol reagent (Life Technologies) and chloroform for phase separation, followed with RNA purification with the RNeasy Mini Kit (Qiagen), and treatment with RNase-Free DNase (Qiagen). DNA-free RNA (1 µg) was reverse transcribed using iScript Reverse transcription (Bio-Rad Laboratories) and gene expression was measured by qPCR using SYBR Green SuperMix (Bio-Rad Laboratories). Final primer concentrations were 0.250 µM for each reaction, and data were analyzed via the CFX Connect Real-Time PCR Detection System (Bio-Rad). The expression of individual genes was normalized to housekeeping gene β-actin and presented as fold-change using the 2^ΔΔCt^ algorithm. Primer sequences are listed in Supplementary Table 1 (see the section on supplementary materials given at the end of this article).

### Histology

Tissues were placed in cassettes and fixed in 10% formalin for 48 h following collection. Tissues were then washed twice with PBS and transferred to 70% ethanol at 4°C for storage before paraffin embedding. All tissues were sectioned at 5µM and transferred to slides at the Research Histology and Tissue Imaging Core of the University of Illinois at Chicago. Tissue sections were stained with hematoxylin and eosin (H&E) using a commercially available staining kit (Vector Laboratories, Burlingame, CA, USA). The slides were then mounted with Poly-mount (Polysciences, Inc., Warrington, PA) and covered with coverslips. Images were acquired with a Leica DMi8 microscope (Leica Biosystems).

### Histological assessment of intestinal inflammation

All slides were blinded, and pathological scores for intestinal inflammation were assessed according to the following criteria: scores ranging from 0 to 3 were given for each tissue section, where 0 indicates no change and 3 indicates maximal change (Geboes *et al.* 2000). Parameters scored included architectural changes, chronic inflammatory infiltration, presence of neutrophils, crypt destruction, erosion and ulceration, and edema. Scores for each parameter were totaled to calculate an overall score.

### Cecal SCFA quantification

Cecal content was isolated from the cecal pouch, weighed and snap frozen at −80°C. The samples were then suspended in 200 µl of 50% methanol and vortexed thoroughly before sonication for 20 min. Subsequently the samples were centrifuged at 27.6 ***g*** for 10 min and 30 µL of the supernatant was taken for derivatization. For derivatization, 30 µL of each standard solution or sample supernatant were mixed with 15 µL of 200 mM 3-NPH in 50% aqueous methanol and 15 µL of 120 mM EDC in the same solution. The reaction was allowed to proceed for 30 min at 40°C. The reaction mix was then diluted with 350 µl of 10% methanol. A volume of 30 µL of the diluted reaction solution was mixed with 30 µL of premade stable isotope-labeled standards for LC/MS analysis using an AB SCIEX 6500 QTRAP coupled with Agilent 1290 UPLC system (Agilent Technologies). The analysis was performed by the Mass Spectrometry Core in Research Resources Center of University of Illinois at Chicago.

### Statistical analysis

Data are presented as mean ± s.e.m. All data were compared by either one-way or two-way ANOVA multiple comparisons (GraphPad Prism 8) followed by a Tukey *post hoc* test when applicable or Student’s t test. Differences in the microbiome’s beta diversity were tested by permutational multivariate analysis of variance (PERMANOVA), a permutation-based multivariate analysis of variance to a matrix of pairwise distance to partition the intergroup and intragroup distance (Segata *et al.* 2011).

## Results

### Loss of intestinal FFA2 does not overtly affect metabolic homeostasis in mice fed a standard chow diet

To validate the intestine-specific knockout of *Ffar2* in the Vil-FFA2 mice, gene expression levels were measured in various tissues in which FFA2 is expressed (Supplementary Fig. 1A). We found that *Ffar2* mRNA expression levels within intestinal tissues were significantly reduced (*P* <0.0001) in the Vil-FFA2 mice compared to expression levels in the FFA2^fl/^ ^fl^ littermate control group, while unchanged in the nonintestinal tissues (Supplementary Fig. 1A and B). Notably, some minimal expression of *Ffar2* was observed within the small intestine in the Vil-FFA2 mice, which is likely due to immune cells embedded within the intestinal mucosa. To probe for changes in metabolic homeostasis due to the absence of intestinal FFA2, male Vil-FFA2 mice and FFA2^fl/^ ^fl^ mice underwent a metabolic characterization on standard chow diet from week 6 to week 25 of age (Supplementary Fig. 1C). No differences in either body weight or *ad libitum* or fasting glucose levels were observed between the two groups throughout the study (Supplementary Fig. 1D, E, and F). Furthermore, no differences in glucose tolerance were observed following either an intraperitoneal (Supplementary Fig. 1G) or oral (Supplementary Fig. 1H) glucose bolus. Finally, no differences were observed in either *ad libitum* or fasting insulin levels (Supplementary Fig. 1I). Collectively, these data indicate that no major congenital or time-dependent differences in metabolic homeostasis exist within the Vil-FFA2 mice as a result of their transgenic composition.

### Vil-FFA2 mice are transiently protected from obesity in response to chronic WD consumption

**Figure 1:**
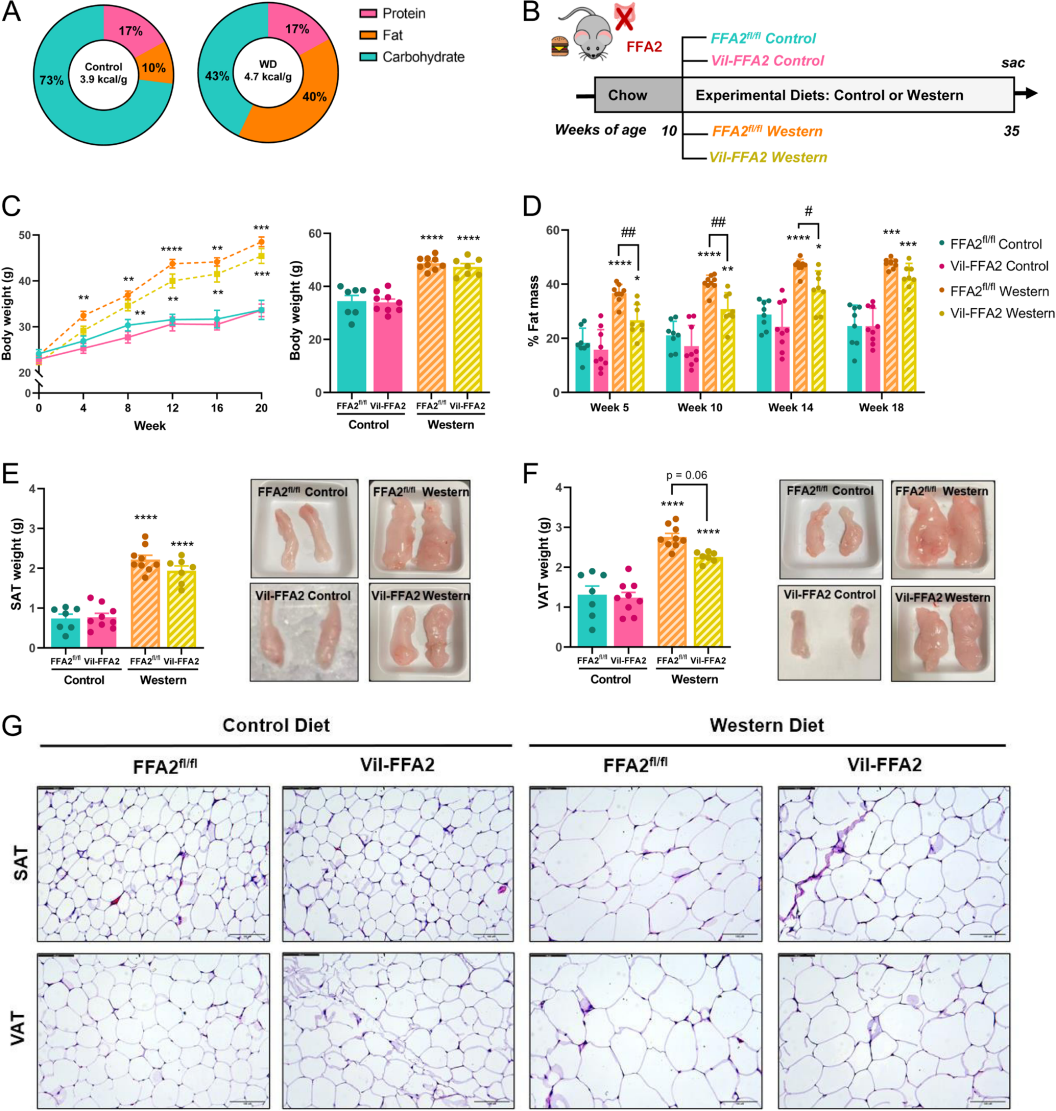
Diet-induced adiposity is significantly reduced in Vil-FFA2 mice. Macronutrient compositions of Western diet (WD) and control diet (CD) (A). Experimental timeline and dietary conditions (B). Body weight measurements during and at the conclusion of the study (C), with week corresponding to the time following dietary intervention. Fat mass represented as a percentage of total body weight (D). Raw weight and images of inguinal subcutaneous (SAT) fat pad (E) and epididymal visceral (VAT) fat pad (F) measured at the conclusion of the study. H&E stain of adipocytes from SAT and VAT (scale bar = 100 µm) (G). For C–G, *n* = 7–9 per group. Values represent mean ± s.e.m., *P < 0.05, **P < 0.01, ***P < 0.001, ****P < 0.0001 vs respective genotypes on CD, and # indicates a significant difference between Western diet groups at ^#^*P* < 0.05, ^##^*P* < 0.01.

Long-term changes in dietary consumption are known to affect the composition of the gut microbiome, altering SCFA levels as one downstream consequence (Ríos-Covián *et al.* 2016). A high-fat, high-sugar ‘Westernized’ style diet has been shown to both reduce levels of SCFAs while accelerating the development of obesity and metabolic stress (Jakobsdottir *et al.* 2013). To determine if the loss of intestinal FFA2 contributes to this process, we performed an obesogenic challenge on male Vil-FFA2 mice and FFA2^fl/^ ^fl^ littermate controls. Mice were either placed on a high-fat, high-sugar Western Diet (WD) or a low-fat control diet (CD) (Fig. 1A). All mice were fed a standard chow until 10 weeks of age and were then placed on either WD or CD and metabolically profiled until 35 weeks of age (Fig. 1B).

Body weights in the WD-fed mice were significantly increased (*P* < 0.01) compared to the CD-fed groups, and this effect was modestly reduced in the WD-fed Vil-FFA2 group, which were on average 3–5 g lighter than the WD-fed FFA2^fl/^ ^fl^ mice (Fig. 1C). Body weights between the WD-fed groups at the end of the study, however, were similar, and both groups developed significant weight gain compared to their CD-fed counterparts (*P* < 0.0001). Measurements of body composition were taken throughout the study using NMR and revealed that the WD-fed Vil-FFA2 group developed significantly less fat mass compared to the WD-fed FFA2^fl/^ ^fl^ control group (*P* < 0.05) (Fig. 1D); however, by 18 weeks into the study, significance was lost. Weights of subcutaneous (SAT) and visceral adipose depots (VAT) at sacrifice after 25 weeks of dietary intervention trended to be lower in the WD-fed Vil-FFA2 group as compared to the WD-fed FFA2^fl/^^fl^ control group (Fig. 1E and F). Additionally, both WD-fed groups had visually large fat depositions compared to the CD-fed groups.

Adipocyte hypertrophy is a hallmark of diet-induced obesity and occurs due to the chronic intake of high levels of dietary fat and sugar, which leads to an energy surplus (Longo *et al.* 2019). To assess for hypertrophy within adipose tissues, fixed and embedded sections were stained for morphology using hematoxylin and eosin. In both subcutaneous and visceral adipocytes, extensive hypertrophy was observed in the WD-fed FFA2^fl/^^fl^ group compared the CD-fed groups (Fig. 1G), visualized by visibly larger droplet size (Murano *et al.* 2008). Adipocyte hypertrophy was present in the WD-fed Vil-FFA2 group, but to a lesser extent, and droplet size was markedly reduced in the subcutaneous adipocytes, while only mildly reduced in the visceral adipocytes, as compared to WD-fed FFA2^fl/^^fl^ group. While the gain of adiposity was roughly equal at the end of the study, when tissue sections were collected, clear differences in the underlying structure of the adipose tissues were maintained. In addition to the reduction in obesogenic diet-induced hypertrophy of both SAT and VAT when FFA2 is absent in the intestine, WD-fed Vil-FFA2 were significantly protected from obesity during the first half of the study, and this effect diminished after 14–16 weeks of dietary intervention. This may indicate a compensatory effect that develops in the absence of intestinal FFA2, allowing the body to accrue fat mass in response to an obesogenic diet. These results suggest that intestinal FFA2 may mediate the genesis of obesity and adipogenicity that occurs as a consequence of long-term consumption of a Western diet.

### WD-fed Vil-FFA2 mice have improved respiratory exchange ratio and lower energy expenditure

**Figure 2:**
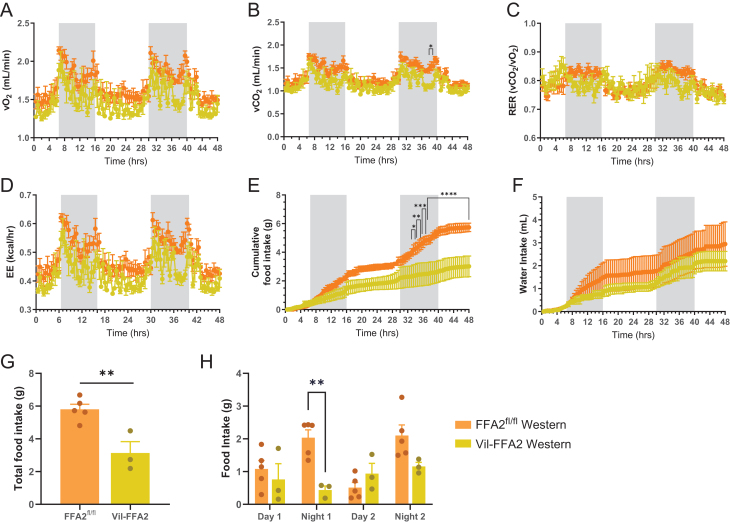
Respiratory gas exchange, energy expenditure, and food intake are altered in the WD-fed Vil-FFA2 mice. Oxygen consumption (A), carbon dioxide production (B), respiratory exchange ratio (RER) (C), energy expenditure (D), cumulative food intake (E), and cumulative water intake (F) measured over a 48-h period in singly housed metabolic cages following 10 weeks of dietary intervention. Cumulative levels of food intake (G) and food intake totals during each day and night of the study (H). Measurements are from WD-fed groups only. *n* = 3–6 per group. Values represent mean ± s.e.m., *P < 0.05, **P < 0.01, ***P < 0.001, ****P < 0.0001 indicates a significant difference between Western diet groups.

Alterations of core metabolic processes including food intake, energy expenditure, and fuel utilization preference can influence body composition and adiposity in a state of energy surplus (Ono-Moore *et al.* 2020). To determine if the protection from obesity observed in the WD-fed Vil-FFA2 mice is influenced by changes in these processes, indirect calorimetry was assessed in mice using metabolic chambers. Mice underwent testing following 10 weeks of dietary intervention, and Western diet groups and control diet groups were measured separately. While oxygen (O_2_) consumption and carbon dioxide (CO_2_) production were unaffected by loss of intestinal FFA2 in the CD-fed groups (Supplementary Fig. 2A and B), the WD-fed Vil-FFA2 mice had significantly lower (*P* < 0.05) respiratory exchange ratio (Fig. 2A and B). To account for differences in body weight that may be contributing to these processes, ANCOVA analysis was performed using total body mass as the covariant (Table 1). Significant differences in lower respiratory exchange ratio processes were preserved after ANCOVA analysis (*P* < 0.05). Consistent with this, respiratory exchange ratio (RER) was also reduced in the WD-fed Vil-FFA2 mice (Fig. 2C), with the most significant reduction occurring during the nocturnal period (*P* < 0.001). Both WD-fed mouse groups had lower RER values throughout the recording period than the CD-fed groups, which would indicate an increase preference for fat oxidation, whereas higher RER values of the CD-fed groups are indicative of a preference to oxidize carbohydrates as source of energy (Supplementary Fig. 2C). Energy expenditure was also found to be reduced in the WD-fed Vil-FFA2 groups compared to the WD-fed FFA2^fl/^ ^fl^ group (*P* < 0.05) (Fig. 2D and Table 1), while unchanged in the CD-fed groups (Supplementary Fig. 2D). Finally, food intake was found to be significantly reduced (*P* < 0.0001) in the WD-fed Vil-FFA2 group compared to the WD-fed FFA2^fl/^ ^fl^ group (Fig. 2E and G), and this effect was not observed in the CD-fed groups (Supplementary Fig. 2E). Water intake was also reduced in the WD-fed Vil-FFA2 group, but not the CD-fed Vil-FFA2 group (Fig. 2F and Supplementary Fig. 2F). In the WD-Vil-FFA2 group, food intake was found to be most significantly reduced (*P* < 0.01) during the night, when mice typically consume the majority of their caloric intake (Fig. 2H) (Ellacott *et al.* 2010). While clear differences in food intake were observed in the WD-fed Vil-FFA2 mice, these changes failed to reach significance in the ANCOVA analysis (Table 1), which identified significant (*P* < 0.05) contributions of body weight to the difference in food intake observed in this dataset. Nonetheless, these data indicate that the absence of intestinal FFA2 results in changes to major metabolic processes in response to chronic consumption of an obesogenic diet. These changes are subsequently reflected in the lower body weight and reduced adiposity observed in the WD-fed Vil-FFA2 mice.

**Table 1:** Metabolic parameters analyzed via ANCOVA.

Effect	Full Day			Light			Dark		
	Mass	Group	Interaction	Mass	Group	Interaction	Mass	Group	Interaction
Food consumed (kcal/period)	0.0491^a^	0.7299		0.1679	0.3761		0.1275	0.299	
Water consumed (mL/period)	0.1034	0.3522		0.4096	0.8760		0.0669	0.2404	
Energy Expenditure (kcal/period)	0.0087^b^	0.0160^a^		<0.001^c^	0.033^a^	0.0251^a^	0.1569	0.0181^a^	
Oxygen consumption (mL/h)	0.0072^a^	0.0161^a^		<0.001^c^	0.0302^a^	0.0224^a^	0.1608	0.0222^a^	
Carbon dioxide production (mL/h)	0.0072^a^	0.0164^a^		<0.001^c^	0.0231^a^	0.0198^a^	0.0794	0.0053^b^	

ANCOVA analysis performed using total body mass as the covariate. *n* = 3–6 per group. * indicates a significant difference between Western diet groups at *P < 0.05, **P <0.01, ***P <0.001.

### Food intake is significantly reduced in the WD-fed Vil-FFA2 mice

**Figure 3:**
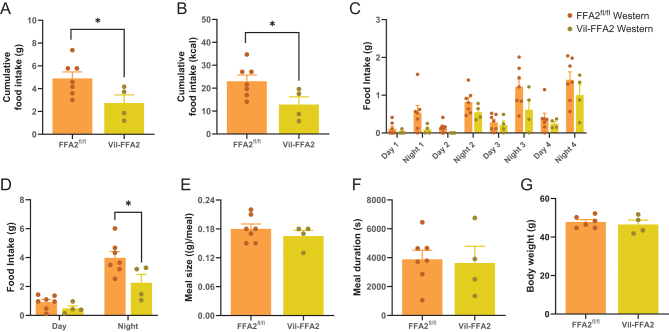
Food intake is significantly reduced in the WD-fed FFA2-Vil mice. Cumulative food intake measured in grams consumed (*A*) and kcal consumed (*B*). Food intake divided into day and night segments (*C*) and time of day (*D*). Meal size (*E*) and meal duration (*F*). Body weights of mice taken at the start of the recording period. Measurements are from WD-fed groups only. *n* = 4–7 per group. Values represent mean ± s.e.m., *P < 0.05 indicates a significant difference between Western diet groups.

To further investigate the reduction in food intake in the WD-fed Vil-FFA2 mice, we performed an identical obesogenic challenge on an additional cohort of mice, consisting of WD-fed groups only. After 20 weeks of dietary intervention, mice were acclimated in singly housed cages designed to sensitively measure food intake. Following a 5-day acclimation period, mice were placed in food-monitoring cages for 96 h and were provided with access to food and water *ad libitum*. We found that cumulative food intake was significantly lower (*P* < 0.05) in the WD-fed Vil-FFA2 group in terms of both grams of food consumed (Fig. 3A) and kcal consumed (Fig. 3B). Consistent with previous data, food intake was also found to be significantly reduced (*P* < 0.05) during the nocturnal hours (Fig. 3D). We also found that despite acclimation to the food intake cages, both groups of mice consumed less food in general, and the amount of food consumed gradually increased as mice adjusted to the cages. Meal size was not different between the two groups (Fig. 3E). Meal duration time also did not differ (Fig. 3F). Body weights were similar between the Vil-FFA2 group and FFA2^fl/^ ^fl^ (Fig. 3G), which was expected given that the experiment took place near the end of the obesogenic challenge. This dataset confirms that the reduction in food intake observed in the WD-fed Vil-FFA2 mice is not due to the reduction in body weight, which was an additional variable to be considered in the metabolic cage dataset.

### In the absence of intestinal FFA2, glucose homeostasis is improved during an obesogenic challenge

FFA2 is a known contributor to the maintenance of glucose homeostasis and has been shown to enhance glucose-stimulated insulin secretion within the pancreatic beta cell (Priyadarshini *et al.* 2015, Villa *et al.* 2016). To determine if the loss of intestinal FFA2 affects glucose metabolism, we probed for glucometabolic differences in the Vil-FFA2 mice.

**Figure 4:**
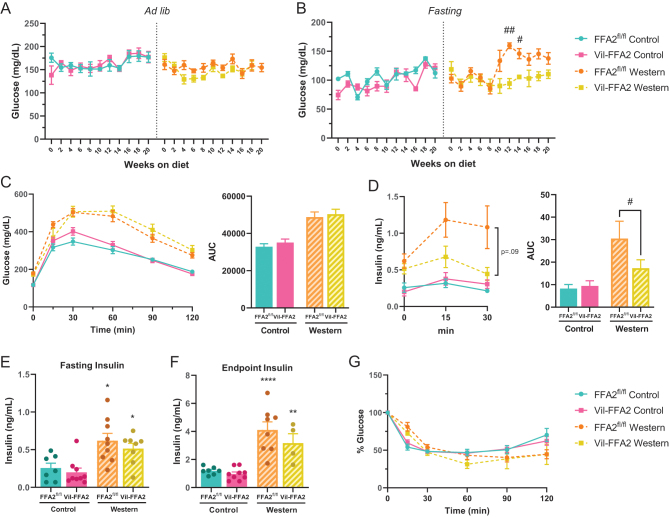
Fasting glucose and glucose-stimulated insulin levels are lower in Vil-FFA2 mice, with no change in glucose sensitivity or insulin tolerance. *Ad libitum* (A) and fasting (B) glucose levels measured throughout the study. O-GTT measured after 14 weeks on respective diet and corresponding AUC (C). Glucose-stimulated insulin levels sampled at 0 min, 15 min, and 30 min during O-GTT and corresponding AUC (D). levels. Fasting plasma insulin levels obtained after 18 weeks on diet (E) and from plasma collected 15 min into an O-GTT at study endpoint (F). ITT measured after 12 weeks on diet (G). For A–G, *n* = 7–10 per group. For *H, n* = 4–8 per group. Values represent mean ± s.e.m., *P < 0.05, **P < 0.01, ****P < 0.0001 vs respective genotypes on CD, and # indicates a significant difference between Western diet groups at ^#^*P* < 0.05, ^##^*P* < 0.01

There were no differences in *ad libitum* glucose levels between the Vil-FFA2 and FFA2^fl/^ ^fl^ mice fed either WD or CD measured throughout the study (Fig. 4A). After 10 weeks on their respective diets, fasting glucose levels in the WD-fed Vil-FFA2 group began trending lower than the WD-fed FFA2^fl/^ ^fl^ group, but only reached significance (*P* < 0.05) during weeks 12–14 of the study (Fig. 4B). Results from an oral glucose tolerance test (O-GTT) performed at week 14 of the study demonstrated that both WD-fed groups developed glucose intolerance when compared to the CD-fed groups, but there was no difference between the Vil-FFA2 and FFA2^fl/^ ^fl^ groups (Fig. 4C). Insulin levels measured at 15 min and 30 min post-glucose bolus during the O-GTT revealed that the WD-fed Vil-FFA2 mice had significantly (*P* < 0.05) lower insulin levels compared to the WD-fed FFA2^fl/^ ^fl^ group, indicating an improvement in glucose-stimulated insulin secretion (GSIS) (Fig. 4D). A modest, but not statistically significant trend of lower insulin levels in the WD-fed Vil-FFA2 group was also observed in fasting insulin levels measured at week 18 of the study (Fig. 4E), and in insulin levels measured 15 minutes into an O-GTT at the end of the study (Fig. 4F), indicating the loss of the previously observed lower GSIS in the WD-fed Vil-FFA2 group. Results from an insulin tolerance test (ITT) on the mice on week 12 of the study revealed no changes in insulin tolerance in any of the groups (Fig. 4G). These data indicate a mild improvement in glucose homeostasis in the WD-fed Vil-FFA2 mice, as indicated by lower fasting glucose levels and lower glucose-stimulated insulin levels; however, there was no difference in glucose tolerance or insulin sensitivity. These data suggest that the absence of intestinal FFA2 has downstream effects on glucose homeostasis in response to an obesogenic challenge and raise the possibility of an intestine-specific contribution of FFA2 to whole-body glucose metabolism. Furthermore, the early emergence and subsequent disappearance of metabolic differences in the WD-fed groups at the end of the study supports the hypothesis that compensatory changes may have developed in response to the ablation of intestinal FFA2 in order to metabolically adapt to an obesogenic challenge.

### Intestinal inflammation and function are unaltered in WD-fed Vil-FFA2 mice

**Figure 5:**
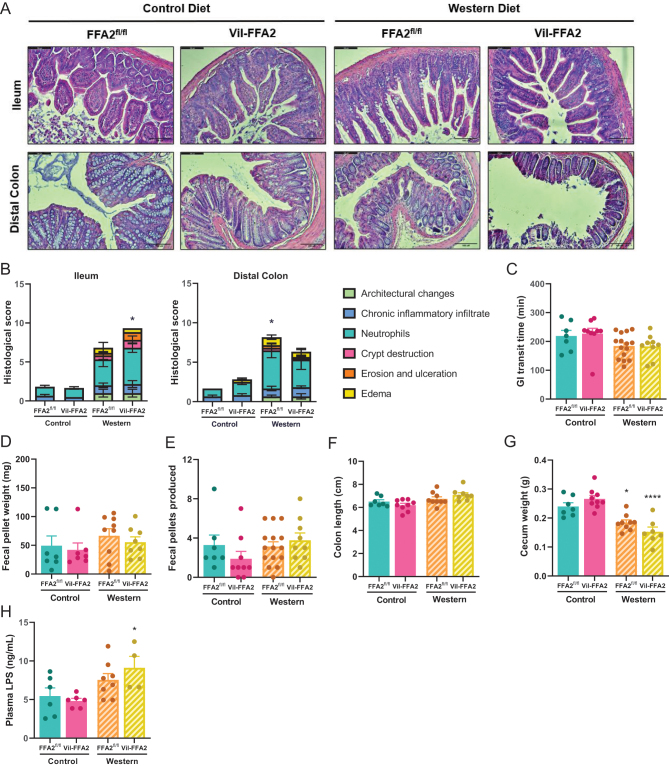
Markers of intestinal inflammation do not differ in WD-fed Vil-FFA2 mice. Representative micrographs of H&E-stained ileum and distal colonic tissues (scale bar = 100 μm) (A). Graphical representation of the average histopathological score for inflammation (B). GI-transit time as measured via carmine red dye assay after 15 weeks on respective diets (C) and fecal pellet weight (D) and number of fecal pellets produced during the first 120 min of the assay (E). Colon length (F), cecum weight (G) and plasma LPS levels (H) measured at the conclusion of the study. For B, *n* = 3–6 per group. For C–G, *n* = 7–15 per group. For H, *n* = 4–8 per group. Values represent mean ± s.e.m., *P < 0.05, ****P < 0.0001 vs respective genotypes on CD.

To determine if the loss of intestinal FFA2 influences intestinal inflammation during obesity, we examined the morphology of H&E-stained sections of both the ileum and distal colon and scored all sections for intestinal inflammation using blinded histopathological scoring. Both WD-fed groups exhibited severe signs of intestinal inflammation, including the infiltration of immune cells, architectural changes, and edema of the underlying lamina propria (Fig. 5A) compared to the CD-fed groups. Interestingly, markers of intestinal inflammation, reflected in histopathological scoring, were not affected by loss of intestinal FFA2 (Fig. 5B) in either the ileum or the distal colon. Overall levels of inflammation were much higher in the distal colons of both WD-fed groups, with no significant difference between the genotypes.

Inflammation is a key component of diet-induced obesity, driving major changes in metabolic pathways throughout the body (Lee *et al.* 2013, Ellulu *et al.* 2017). Severe inflammation can substantially impair the functionality of the intestine, affecting processes including transit time, permeability, and nutrient absorption, which may in turn contribute to alterations in body weight (Peuhkuri *et al.* 2010). To assess the functionality of the intestine during the loss of intestinal FFA2, we first performed a GI-TT on all mouse groups following 14 weeks of dietary intervention. While GI-TT trended modestly lower in both WD-fed groups (Fig. 5C), there were no differences between the Vil-FFA2 group and FFA2^fl/fl^ group on either diet. Fecal measurements indicative of severe diarrhea, including fecal pellet production (Fig. 5E) and pellet weight (Fig. 5D) were unaffected by either diet or genotype. Intestinal permeability, measured by the leaking of endotoxin LPS into the systemic plasma, was found to be significantly higher (*P* < 0.05) in the WD-fed Vil-FFA2 group compared to the CD-fed Vil-FFA2 group (Fig. 5H), and plasma LPS levels were elevated in both WD-fed groups. However, no major differences between the WD-fed groups were observed, indicating that elevated plasma levels are likely due to the change in diet. Other features of intestinal inflammation, such as shortening of the colon (Fig. 5F) and decreased weight of cecal content (Fig. 5G), were unaffected by the loss of intestinal FFA2; however, both WD groups had significantly lighter cecal contents. These data indicate that while WD-feeding produced a mildly dysfunctional intestine, alterations in gut function due to the loss of intestinal FFA2 are not driving the phenotypic differences observed in the Vil-FFA2 mouse group.

FFA2 is thought to mediate the secretion of several postprandial gut peptide hormones, though data are largely reliant on the use of global KO models of the receptor and is often conflicting (Psichas *et al.* 2005, Akiba *et al.* 2017). To determine if* in vivo* incretin secretion is altered in response to an oral glucose challenge, we measured incretin levels in the plasma from mice taken 15 min into an O-GTT at the end of the study. GLP-1 levels were significantly elevated (*P* < 0.05) when compared to the CD-fed group, and GIP levels also trended higher in the WD-fed groups, indicating an altered incretin profile in response to an obesogenic challenge (Supplementary Fig. 3). However, we observed no change in GLP-1 levels (Supplementary Fig. 3A) nor GIP levels (Supplementary Fig. 3B) between the WD-fed groups. These data indicate that there is no difference in glucose-stimulated GLP-1 or GIP release in the WD-fed Vil-FFA2 group that may be contributing to phenotypic changes. However, these measurements were taken at the end of the study and therefore are not sufficient to conclude that there are no alterations in incretin secretion at prior time points.

### Intestine-specific deletion of FFA2 alters the gut microbiome, cecal SCFA levels, and expression of their cognate receptors

**Figure 6:**
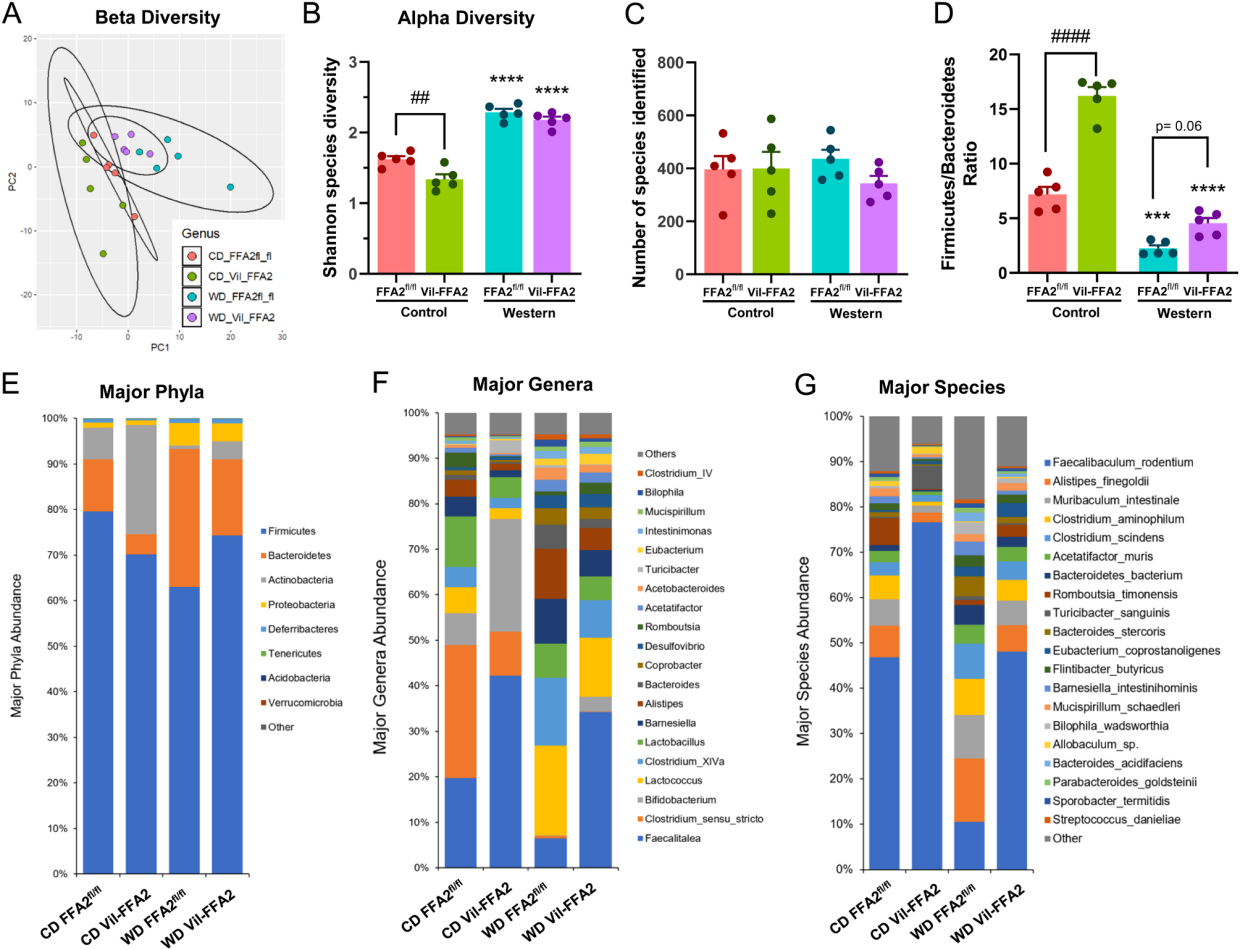
Intestine-specific deletion of FFA2 alters the fecal gut microbiome. Beta diversity as indicated by principal component analysis (PCA) (A) and alpha diversity as indicated by Shannon index (B). Total number of unique species identified (C) and ratio of Firmicutes to Bacteroidetes (D). Relative abundance of major phyla (E), relative abundance of major genera (F) and species (G). Values represent mean ± s.e.m., *n* = 5 per group, ***P < 0.01, ****P < 0.0001 vs respective genotypes on CD and # indicates a significant difference between genotypes in the same diet group at ^##^*P* < .01, ^####^*P* < 0.0001.

Diet-induced variation in the composition of the gut microbiome is suggested to play a key role in the pathogenesis of obesity, with numerous studies identifying distinct differences in bacterial phyla as well as differences in diversity and richness in obese individuals when compared to lean individuals (Cotillard *et al.* 2013). Consumption of a ‘Western’ style diet, rich in processed fats and sugars while low in fibers, has been shown to alter the abundances of bacteria in the Firmicutes, Bacteroidetes, and Proteobacteria phyla, shifting the microbial community toward metabolizers of simple sugars while decreasing bacterial species associated with the production of SCFA and other metabolites (Bolte *et al.* 2021). To understand how changes in the gut microbiota influence phenotypic differences in the absence of intestinal FFA2, 16S sequencing was performed on fecal samples from all experimental mouse groups. Beta diversity, which is the measurement of similarity between microbial communities, revealed unique clustering among the WD-fed groups compared to the CD-fed groups (Fig. 6A). The effect of diet on the diversity of the gut microbiome produced a much greater effect than genotype. While consumption of WD modestly increased the Shannon species index in both WD-fed groups, a difference in microbial diversity between the Vil-FFA2 and FFA2^fl/^ ^fl^ mice was only observed in the CD-fed groups (*P* < 0.01) (Fig. 6B). The total number of bacterial species identified was similar irrespective of either diet or genotype (Fig. 6C). Interestingly, the Firmicutes–Bacteroidetes ratio significantly increased (*P* < 0.0001) in the CD-fed Vil-FFA2 group compared to the CD-fed FFA2^fl/^ ^fl^ controls, but only modestly in the WD-fed Vil-FFA2 group compared to the WD-fed FFA2^fl/^ ^fl^ group (Fig. 6D). A higher Firmicutes–Bacteroidetes ratio typically signifies a less healthy gut; however, high levels of Actinobacteria (Fig. 6E) in the CD-fed groups in addition to high abundance of Proteobacteria likely skewed these results in the WD group.

Alterations in bacterial genera were also observed in the WD-fed groups compared to the CD-fed groups, and WD consumption was found to decrease *Bifidobacterium*, *Acetobacteroides, Turicibacter*, and *Lactobacillus* while increasing *Clostridium* cluster XVIa*, Lactococcus, Barnesiella*, and *Alistipes* (Fig. 6F). At the species level, all groups except the WD-fed FFA2^fl/^ ^fl^ group had a high abundance of *Faecalibaculumrodentium* (Fig. 6G). While unique signatures of each mouse group were observed, LefSe analysis could not be performed because these changes did not meet the significance threshold. These changes indicate that consumption of WD alters the gut microbial composition relative to consumption of CD. Furthermore, deletion of intestinal FFA2 resulted in significant changes within the CD-fed groups. Interestingly, while differences between the WD-fed FFA2^fl/^ ^fl^ group and WD-fed Vil-FFA2 group were observed, these changes appeared more pronounced between the CD-fed groups.

**Figure 7:**
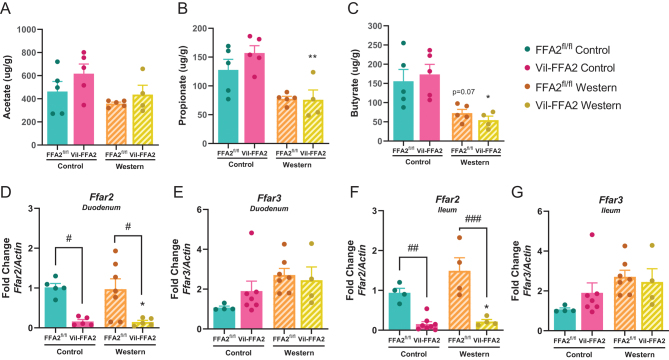
Cecal SCFA levels and expression of intestinal SCFA receptors are influenced by dietary changes in Vil-FFA2 and FFA2^fl/fl^ mice. Levels of SCFAs (A) acetate, (B) propionate, and (C) butyrate as measured by GC-MS. (B). Expression of Ffar2 (D) and Ffar3 (E) in the ileum. (F, G) Expression of Ffar2 (F) and Ffar3 (G) in the distal colon. Values represent mean ± s.e.m. For A-C,* n* = 4–5. per group. For D–G, *n* = 4–7 per group, *P < 0.05, **P < 0.01 vs the CD-fed FFA2^fl/fl^ control group and # indicates a significant difference between genotypes in the same diet group at ^#^*P* < 0.05, ^##^*P* < 0.01, ^###^*P* < 0.001.

SCFAs are the endogenous ligands for FFA2 and are predominantly generated in the distal colon before absorption by the intestinal epithelium (Priyadarshini *et al.* 2021). Therefore, we assessed how diet, in addition to the absence of FFA2, would affect cecal SCFA levels. Consistent with literature reporting reduced SCFA levels in a state of gut dysbiosis (Wang *et al.* 2014, Agus *et al.* 2016), we observed reduced levels of acetate, propionate (*P* < 0.05) and butyrate (*P* < 0.01) in both WD-fed mouse groups, with no differences between the Vil-FFA2 and FFA2^fl/^ ^fl^ groups (Fig. 7A, B, and C). These data also suggest that differences in SCFA production are not driving the phenotypic changes observed in the WD-fed Vil-FFA2 mice.

Finally, we examined changes in the intestinal gene expression levels of SCFA receptors in response to diet as well as ablation of intestinal FFA2. Of importance, FFA2, along with its highly similar sister receptor, FFA3, comparatively possesses the highest affinity for SCFAs (Schmidt *et al.* 2011). Within both the duodenum and ileum of the intestine, FFA2 was, as expected, significantly (*P* < 0.05) ablated in both Vil-FFA2 groups irrespective of diet (Fig. 7D) and expression was similar between the CD and WD-fed FFA2^fl/^^fl^ groups. FFA3 trended higher in the CD-fed Vil-FFA2 group compared to the CD-fed FFA2^fl/^^fl^ control group, and both WD-fed groups contained equally elevated expression levels that were modestly higher (Fig. 7E, F, and G). This indicates that the absence of intestinal FFA2 does not significantly influence expression of FFA3, suggesting that FFA3 is not driving the compensatory effect observed in the WD-fed Vil-FFA2 group during second half of the obesogenic challenge.

## Discussion

FFA2 is an important mediator of the metabolic effects of SCFAs, both within the digestive system and throughout the body, acting through a summation of its many tissue-specific effects. FFA2 is highly expressed in the intestinal epithelium, where it has been reported to contribute to metabolic homeostasis primarily by influencing the secretion of gut peptide hormones (Psichas *et al.* 2005, Karaki *et al.* 2006, Dass *et al.* 2007, Tolhurst *et al.* 2012, Kimura *et al.* 2013). Previously, our group characterized a novel, intestine-specific knockout mouse model for FFA3 in response to an obesogenic challenge, revealing novel insights into receptor function (Lednovich *et al.* 2022). In this study, we utilize a similar strategy to explore the intestine-specific roles of FFA2 by generating the Vil-FFA2 strain of mice. Here, we report the first *in vivo* characterization of intestinal FFA2 in response to two distinct diets—standard chow, and a high-fat, high-sugar Western diet. We found that WD-fed Vil-FFA2 mice were largely protected from the development of obesity and hyperglycemia during the initial stage of dietary intervention, but this protection was lost by the end of the study. WD-fed Vil-FFA2 mice had visibly less hypertrophy of adipocytes in both subcutaneous and visceral adipose tissues, despite both groups having the same body weight and similar fat mass at the end of the study. Analysis of indirect calorimetry revealed that the decrease in adiposity in the WD-fed Vil-FFA2 mice is influenced by changes in core metabolic processes, including respiratory exchange ratio and energy expenditure, and these changes likely occur as a result of an underlying decrease in food intake. Collectively, these data indicate that intestinal FFA2 contributes to the genesis of obesity during acute consumption of an obesogenic diet.

While no overt changes in whole-body physiology or metabolic parameters were detected in mice fed a standard chow diet, we observed significant alterations that occurred in mice lacking intestinal FFA2 when fed WD. During the first 15 weeks of the obesogenic challenge, WD-fed Vil-FFA2 mice had significantly lower body weight and fat mass compared to the WD-fed FFA2^fl/^ ^fl^ control mice. Interestingly, this trend appeared to be transient, and was the most apparent early in the study before gradually disappearing by the study’s endpoint. While the WD-fed mouse groups had similarly sized adipose depositions at the conclusion of the study, markers of hypertrophy – a hallmark of obesity – were reduced in the WD-fed Vil-FFA2 mice. These observations indicate that the loss of intestinal FFA2 results in the transient protection of the obesogenic consequences of WD consumption, but physiological compensatory mechanisms may emerge to correct these differences, allowing the body to accumulate fat mass in the absence of the receptor. However, the persistence of reduced adipose hypertrophy in the Vil-FFA2 mice suggests that these compensatory effects are insufficient to fully ameliorate the metabolic changes driven by the loss of the receptor in the intestine.

Changes in body weight have been previously reported in studies utilizing global knockout models for FFA2, although conflicting results have been reported. Several studies utilizing global knockout mouse models for FFA2 under obesogenic conditions report a higher proportion of fat mass and increased body weights while other groups report the opposite effect (Bjursell *et al.* 2011, Kimura *et al.* 2013, Forbes *et al.* 2015, Brooks *et al.* 2017). Building off of the previous observation that global ablation of FFA2 results in alterations in body weight during an obesogenic challenge, we identify that these changes occur predominantly by differences in fat mass and that intestinal FFA2 contributes to this process. Furthermore, our data reveal that adipocyte hypertrophy in both subcutaneous and visceral adipose tissues is reduced in the absence of intestinal FFA2 even when there are no significant differences in overall body weight. Collectively, these data suggest a role for intestinal FFA2 in mediating the obesogenic effects of consumption of a Western diet.

The loss of intestinal FFA2 results in significant changes to core metabolic processes when body weight was considered as an independent variable. Collectively, our data indicate that the WD-fed Vil-FFA2 mice are metabolizing more fat and have reduced food intake, which subsequently could impact energy expenditure. While there are no reports on the effect of FFA2 on RER or energy expenditure in other literature, these findings are in agreement with a reduced adiposity during the obesogenic challenge in this study.

Food intake has a major impact on downstream metabolism, including energy homeostasis, driving whole-body changes in adiposity and glycemic control (Westerterp 2016). We observed that the WD-fed Vil-FFA2 mice had reduced nocturnal food intake during multiple time points in the study, and this effect persisted despite the transient nature of the reduction in body weight and improved glycemic control. FFA2 has been previously implicated in the regulation of feeding behaviors. One study observed that global FFA2 KO mice fed with a high-fat diet (HFD) developed increased food intake but lower overall body weight (Bjursell *et al.* 2011). A similar study performed with the addition of fiber observed a similar effect on satiety (Brooks *et al.* 2017). These effects were attributed, in part, due to changes in PYY secretion, which has a known effect on postprandial satiety. While we report the opposite effect – that loss of intestinal FFA2 results in a decrease in satiety – this discrepancy may be the result of using an intestine-specific KO model rather than a whole-body KO model. Furthermore, multiple experimental differences, including different diets, background strains of mice, and microbiome compositions are known variables in studies involving metabolism. Because our study did not ascertain a causative role of food intake on the other metabolic differences observed in the WD-fed Vil-FFA2 mice, nor determine the underlying mechanisms involving the receptor in the intestine, further studies involving feeding behavior will be crucial to fully interpret the metabolic phenotype observed here.

In addition to a decrease in body weight, we observed a modest improvement in glycemic control in the WD-fed Vil-FFA2 group but no changes in glucose tolerance or insulin action. Fasting glucose levels in the WD-fed Vil-FFA2 group trended lower than the WD-fed FFA2^fl/^ ^fl^ mice beginning at 8 weeks the study, and significantly diverged during weeks 12–14 following dietary intervention. Similar to the trend observed in fat mass, statistically significance disappeared by the end of the study despite modestly lower levels of fasting glucose in the WD-fed Vil-FFA2 group. From these results, it is unclear whether the loss of intestinal FFA2 directly contributes to changes in glucose metabolism – which may occur through alterations in the secretion of incretin hormones – or whether these changes are secondary effects caused by differences in fat mass (Peiris *et al.* 1988). The significant reduction in GSIS observed in the WD-fed Vil-FFA2 mice compared to the WD-fed floxed group raises the possibility of changes occurring within the entero-insular axis. While other groups have observed the potential for FFA2 to mediate incretin hormones (Psichas *et al.* 2005, Tolhurst *et al.* 2012), we did not observe any changes in plasma GLP-1 or GIP during a separate GSIS. However, these measurements were collected at the conclusion of the study, when several of the phenotypic differences in the WD-fed Vil-FFA2 mice, including reduced body weight and adiposity, were not present.

FFA2 has been suggested to regulate immunogenic effects through its role in immune cells and also in IECs (Kim *et al.* 2013, Masui *et al.* 2013, Nowarski *et al.* 2015, Kamp *et al.* 2016). While we observed an increase in inflammatory markers in both WD-fed groups, no significant differences were observed between the WD-fed Vil-FFA2 compared to the WD-fed FFA2^fl/^ ^fl^ mice. No major changes in intestinal inflammation were observed through histological analysis of the both the upper and lower intestine, nor were functional changes in gut permeability or transit time observed in the WD-fed Vil-FFA2 mice. While these results suggest that loss of intestinal FFA2 does not affect the body’s ability to mount an inflammatory response during chronic consumption of an obesogenic diet, it is crucial to note that intestinal inflammation was assessed at the end of the study, time at which several major phenotypic differences in the Vil-FFA2 group had disappeared. This underscores the need for additional studies utilizing an acute obesogenic challenge to fully assess phenotypic differences prior to the influence of any potential compensatory mechanism.

There is ample evidence that FFA2 influences the release of postprandial gut peptide hormones within intestinal EECs. While there are some conflicting data regarding which specific hormones are affected, multiple studies have reported that FFA2 is able to promote the release of hormones GLP-1 and PYY in response to SCFA stimulation *in vitro* and *ex vivo*, with GIP and 5-HT also being implicated (Psichas *et al.* 2005, Tolhurst *et al.* 2012, Iwasaki *et al.* 2015, Akiba *et al.* 2017). In this study, we did not find any evidence of altered GLP-1 or GIP levels when measured 15 min into an oral glucose tolerance test, which is when incretin levels typically peak after stimulation with glucose. However, changes in incretin production are notoriously difficult to measure *invivo*, including complex release patterns that produce a state of flux and rapid degradation of hormones following their production (Baggio & Drucker2007, Mentlein *et al.* 2009). Due to these challenges, we performed a number of experiments to measure indirect effects of incretin hormones, specifically GLP-1 and PYY. Alterations in GLP-1, an insulinotropic peptide hormone, may be reflected by changes in glucose levels, insulin levels, glucose tolerance, or food intake (Holst 2007). Alterations in PYY may be reflected by changes in gut motility, energy expenditure, and food intake (Karra *et al.* 2009). We observed a reduction in food intake in the WD-fed Vil-FFA2 mice, which may be driven by changes in incretin production, but more direct evidence is needed to ascertain the involvement of GLP-1 and PYY. Furthermore, food intake is influenced by many other hormones, including cholecystokinin, leptin, somatostatin, and ghrelin (Hajishafiee *et al.* 2019, Kumar & Singh2020).

While we did observe a modest improvement in glucose homeostasis in the WD-fed Vil-FFA2 mice, it is unclear if this effect is due to changes in GLP-1 or GIP secretion. Because FFA2 is thought to stimulate the release GLP-1 from EECs, the absence of the receptor might lead to the opposite observation: an impaired GLP-1 response leading to hyperglycemia and diminished insulin release. Similarly, FFA2 is also thought to stimulate the release of PYY, so the absence of the receptor in the intestine might lead to decreased gut motility and reduced satiety, ultimately resulting in an increase in obesity. Furthermore, GLP-1 and PYY are anorectic hormones, and their loss leads to increased food intake and subsequent weight gain (De Silva *et al.* 2011). The findings in our study, including the reduction of food intake, decreased fat mass and improved glucose metabolism, are incongruent with the phenotype that is expected to be observed with impaired GLP-1 and PYY production. There are several potential reasons for this. Importantly, gut peptide hormones are known to substantially compensate for each other, which has been shown to occur in knockout mouse models for incretin receptors GLP1R and Y2R (Boland *et al.* 2019*a*,* b*). Furthermore, the secretion patterns of gut peptide hormones become altered during the course of chronic Western diet feeding, and likely fluctuated substantially over the course of this study (Wikarek *et al.* 2014). Because of the complexity in measuring the effects of these hormones *in vivo*, future studies utilizing a secretomics approach from intestinal tissues derived from the Vil-FFA2 may be useful in identifying the full spectrum of hormonal changes that occur in response to the loss of intestinal FFA2 and will have additional value in understanding the mechanisms driving the reduction in feeding behavior.

In accordance with other published literature, we found that the gut microbiome is significantly altered in response to chronic consumption of a Western-style diet (Zinöcker & Lindseth2018). Furthermore, changes were influenced by the absence of intestinal FFA2 and interestingly, these changes were more pronounced in the CD-fed groups than the WD-fed groups in the FFA2 study. Consumption of WD produced a significantly different microbial signature when compared to the CD-fed groups. Long-term consumption of WD led to a shift in bacteria phyla, with WD-fed groups containing a higher abundance of Bacteroidetes and Proteobacteria in addition to a decrease in Actinobacteria. While Bacteroidetes is generally considered a beneficial type of bacteria, the significant increase in Proteobacteria may be indicative of a diseased state and is observed in both inflammatory bowel disorders and metabolic disorders (Rizzatti *et al.* 2017). Similarly, Actinobacteria have known roles in carbohydrate metabolism, and their decreased abundance in the WD-fed groups is expected (Binda *et al.* 2018). At the phyla level, the Vil-FFA2 groups seemed to harbor less beneficial bacteria and had more of the detrimental *Firmicutes* bacteria. While changes at the bacterial species level were observed in the WD-fed Vil-FFA2 mice compared to the WD-fed FFA2^fl/^ ^fl^ controls, these changes were largely insignificant. This suggests that differences in the gut microbiome itself are likely not driving the metabolically protective effects observed in the Vil-FFA2 mice. In accordance with these findings, levels of cecal SCFAs were unaltered in the WD-fed Vil-FFA2 mice compared to the WD-fed FFA2^fl/^ ^fl^ control group.

The observation of similar microbial signatures and comparable SCFA levels between the two WD-fed groups, despite distinct metabolic phenotypes, likely reflects the strong shift toward bacteria involved in fat and sugar metabolism, which become necessary to support diet-induced changes. Interestingly, this indicates that the differences in body weight, glucose metabolism and food intake observed in the WD-fed Vil-FFA2 mice do not appear to be directly driven by changes in the gut microbiome-SCFA-receptor axis, or by differences in the production of the endogenous ligands of FFA2. The findings from this study instead supports the hypothesis that FFA2 plays an important role in the presence of metabolic stress, which occurs during an obesogenic state, and has been previously observed in other *in vivo* models involving this receptor (Peuhkuri *et al.* 2010, Kimura *et al.* 2013, Lee *et al.* 2013). Future studies involving this receptor should aim to uncover the exact mechanisms by which diet-induced, metabolic stress effects the function of FFA2.

FFA2 is highly expressed in the intestinal epithelium and contributes maintenance of metabolic homeostasis by mediating the effects of its ligands, microbial-derived SCFAs, throughout the body. Here, we report the first *in vivo* characterization of FFA2 within the intestine and reveal novel insights into receptor function. We found that intestinal FFA2 plays an important role in mediating the obesogenic consequences of a WD, and that alterations in food intake likely drive to this process. Collectively, these data support an intestine-specific role of FFA2 in the regulation of food intake, as well as the maintenance of metabolic homeostasis and in the development of obesity.

## Supplementary materials

Figure S1: Loss of intestinal FFA2 does not overtly affect metabolic homeostasis in mice fed a standard chow diet.

Figure S2: Respiratory gas exchange, energy expenditure and food intake are unaltered in CD-fed Vil-FFA2 mice.

Figure S3: No difference in glucose-stimulated secretion of incretin hormones are observed in the WD-fed Vil-FFA2 mice.

Table S1: List of qPCR Primers

## Declaration of interest

The authors declare no conflicts of interest, financial or otherwise.

## Funding

K.R.L. is supported by the John and Kathy Solaro Graduate Fellowship through the Physiology and Biophysics Department at the University of Illinois College of Medicine. B. T. Layden is supported by National Institutes of Healthhttp://dx.doi.org/10.13039/100000002 under Award Number R01DK104927 and P30DK020595; and Department of Veterans’ Affairs, Veterans Health Administration, Office of Research and Developmenthttp://dx.doi.org/10.13039/100006379, VA merit (Grant No. 1I01BX003382). H.Y. is supported by funding from NIH – R21AG072379; RF1AG071762; R56AG064075, and Department of Defense – W81XWH-18-PRARP-NIRA.

## Author contribution statement

K.R.L., M.P., B.W., H.Y., and B.T.L conceived and designed experiments. K.R.L., C.N., S.G., M.P., N.P., K.X., J.L.Z, S.M., S.J., and B.W. performed experiments. K.R.L analyzed data, interpreted results of experiments, and prepared figures. K.R.L drafted the manuscript. K.R.L, C.N., J.C.C, S.G., M.P., B.W., J.L.Z, H.Y., and B.T.L edited the manuscript, and B.T.L approved the final version.
