# Supplementary figures and images for: Intestinal FFA2 promotes obesity by altering food intake in Western diet-fed mice
Source: J Endocrinol. 2024 Jan 11;260(2):e230184. doi: 10.1530/JOE-23-0184 (PMC10831573; doi:10.1530/JOE-23-0184)

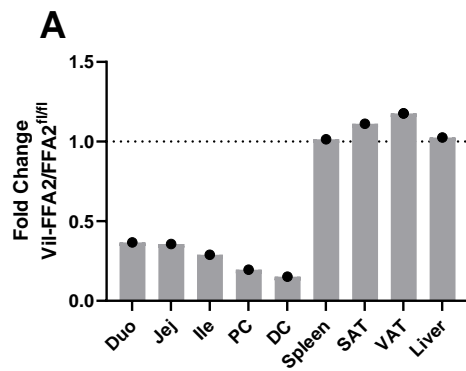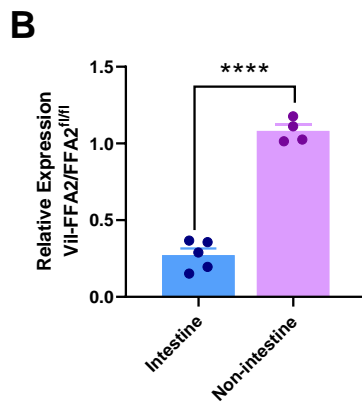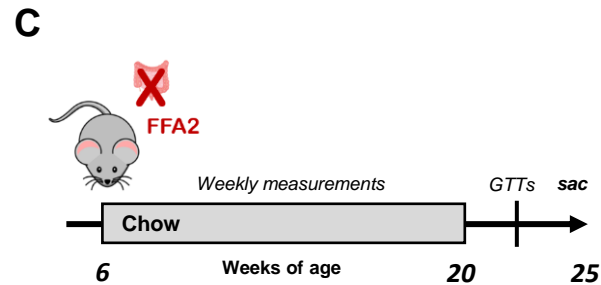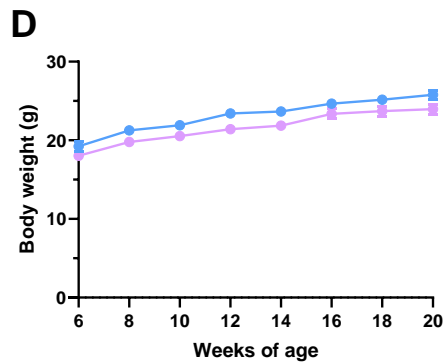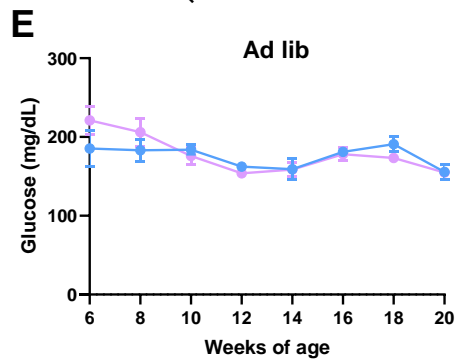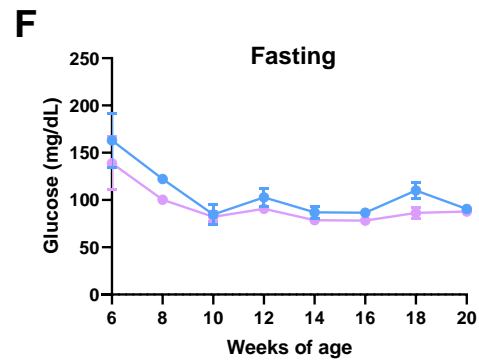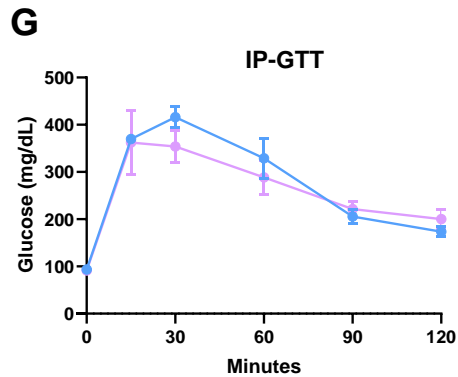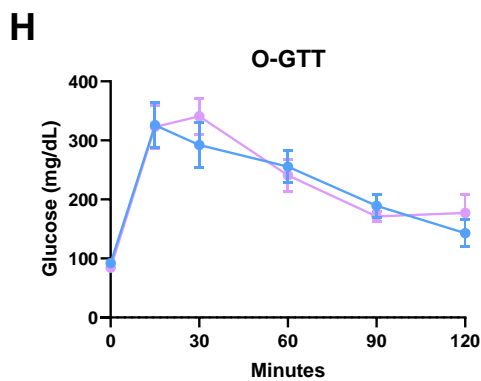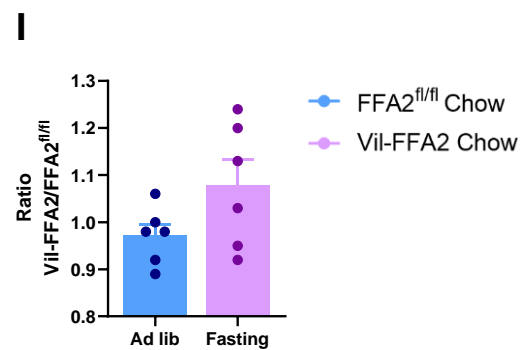

Supplement: Figure S1: Loss of intestinal FFA2 does not overtly affect metabolic homeostasis in mice fed a standard chow diet. [file supplementary_figure_1.pdf]

**A**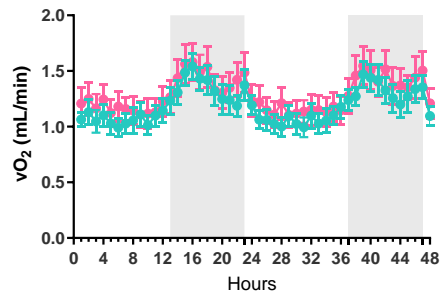**B**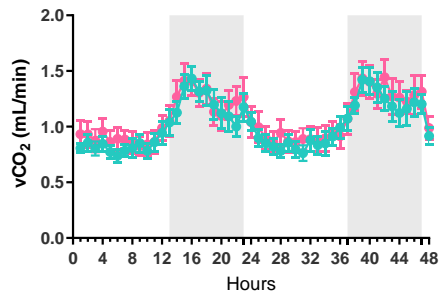**C**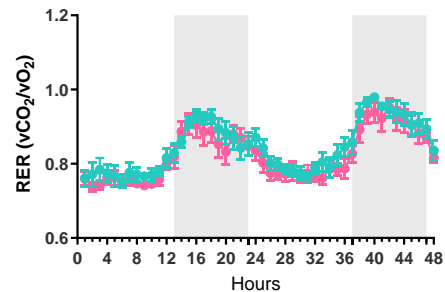**D**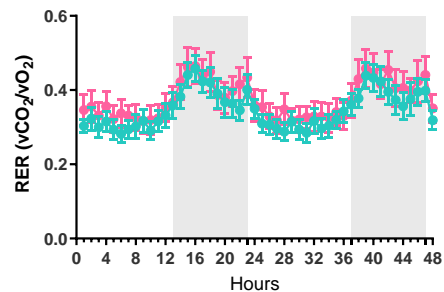**E**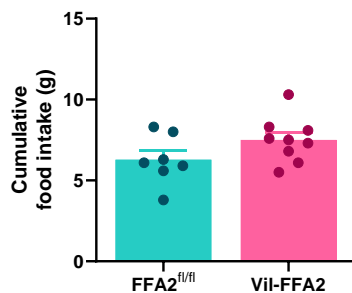**F**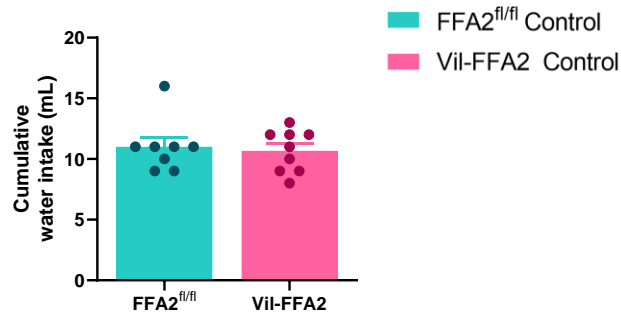

Supplement: Figure S2: Respiratory gas exchange, energy expenditure and food intake are unaltered in CD-fed Vil-FFA2 mice. [file supplementary_figure_2.pdf]

**A**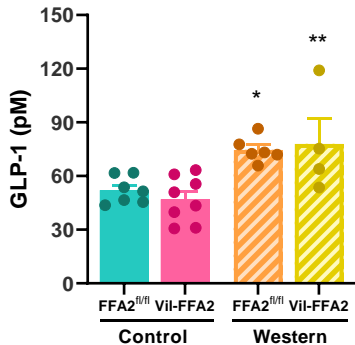**B**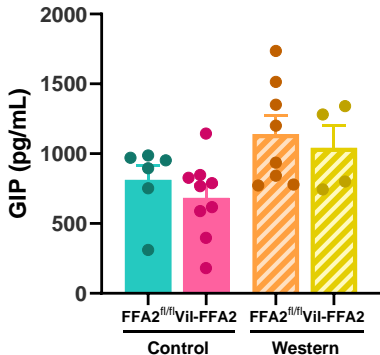

Supplement: Figure S3: No difference in glucose-stimulated secretion of incretin hormones are observed in the WD-fed Vil-FFA2 mice. [file supplementary_figure_3.pdf]
