# Supplementary material for: Intestinal FFA2 promotes obesity by altering food intake in Western diet-fed mice
Source: J Endocrinol. 2024 Jan 11;260(2):e230184. doi: 10.1530/JOE-23-0184 (PMC10831573; doi:10.1530/JOE-23-0184)
Supplement: Table S1: List of qPCR Primers [file supplementary_table_1.pdf]

Table S1: List of qPCR Primers

Gene	Forward Primer 5'-3'	Reverse Primer 5'-3'
Actin	TACCACAGGCATTGTGATGG	TCTCAGCTGTGGTGGTGAAG
Ffar3	CAGTGGCTGTGGACTTACTT	GGAGGTGAGGTAAATAGTGGTG
Ffar2	AGAGAACCAGAGAGGATGTGA	GGAGCCCAGTAAGAAAGATGAG
